# SHQ1 is an ER stress response gene that facilitates chemotherapeutics-induced apoptosis via sensitizing ER-stress response

**DOI:** 10.1038/s41419-020-2656-0

**Published:** 2020-06-10

**Authors:** Huimin Liu, Siqi Xie, Fang Fang, Dhananjaya V. Kalvakolanu, Weihua Xiao

**Affiliations:** 1 Department of Oncology of the First Affiliated Hospital, Division of Life Sciences and Medicine, University of Science and Technology of China, 230027 Anhui Hefei, China; 2 Hefei National Laboratory for Physical Sciences at Microscale, the CAS Key Laboratory of Innate Immunity and Chronic Disease, School of Life Sciences, University of Science and Technology of China, Hefei, China; 3 Institute of Immunology, University of Science and Technology of China, Hefei, China; 4 Engineering Technology Research Center of Biotechnology Drugs Anhui, University of Science and Technology of China, 230027 Anhui Hefei, China; 5 Department of Microbiology and Greenebaum Comprehensive Cancer Center, University of Maryland School of Medicine, Baltimore, MD 21201 USA

**Keywords:** Chemotherapy, Stress signalling

## Abstract

SHQ1 was reported to control the biogenesis and assembly of H/ACA ribonucleoprotein particles (RNPs). It was independently isolated as a growth suppressor, GRIM1, in a genetic screen. Recent studies have indicated that SHQ1 inhibits prostate cancer growth and metastasis. SHQ1 facilitates MYC RNA splicing to promote T-acute lymphoblastic leukemia (T-ALL) development. Thus, the mechanisms of SHQ1 in cancers remain largely unknown. We report here that SHQ1 promotes tumor apoptosis and chemo-sensitivity in hepatocellular carcinoma (HCC) cells. In HCC tissues from patients, expression of SHQ1 was significantly decreased in the tumor compared to adjacent tissues. Experiments with HCC xenograft models revealed that restoring SHQ1 levels enhanced the anti-tumor activity of the endoplasmic reticulum (ER) stress inducer tunicamycin (TM) and common chemotherapy drug paclitaxel (PTX). Mechanistically, SHQ1 is an ER-stress response gene which is regulated by p50ATF6 and XBP1s through an ER stress response like element located on the SHQ1 promoter. SHQ1 interacts with the ER chaperone GRP78 to release ER sensors PERK/IRE1*α*/ATF6 from GRP78/ER-sensor complexes, leading to hyper-activation of unfolded protein response (UPR). In the persistent ER stress conditions of a HepG2 xenograft tumor model, SHQ1-mediated hyper-activation of ER-sensor signaling induces apoptosis. Our study thus demonstrates a SHQ1-mediated ER-stress response feedback loop that promotes tumor sensitivity to chemotherapeutics.

## Introduction

Given the evidence that SHQ1, also named GRIM1, is isolated as a growth suppressor using genetic approaches^1^. SHQ1 codes for a human orthologue of yeast protein, Shq1p. Human SHQ1 contains an N-terminal CS domain that is similar to Shq1p and a C-terminal Shq1-specific domain (SSD)^2^. A serine to proline substitution in the CS domain of SHQ1 is associated with some prostate cancers^2^. The C-terminal region of human SHQ1 is rich in serine/threonine residues unlike the Shq1p^3^. SHQ1 suppresses box H/ACA RNA levels through sequestering NAF1 which is required for box H/ACA sno/sca RNP biogenesis in mammalian cells^3^. Integrative genomic profiling reveals that the frequency of SHQ1 deletion in chromosome 3p of prostate cancer and cervical invasive carcinomas are 14.7% and 61%, respectively^4,5^. SHQ1 cooperates with PTEN to inhibit the development and metastasis of prostate cancer in mice^6^. The recurrence of prostate cancer is associated with the loss of *PTEN* and 3p13 locus spanning *FOXP1* to *SHQ1*^7–9^. Deletion of 3p13 is closely linked to ERG fusion-positive prostate cancer and *PTEN* deletion^4^. Isolated deletion of 3p13 and co-deletion with *PTEN* are 16.5% and 7.7%, respectively, in ERG fusion-positive prostate cancer^4^. A recent study have indicated that SHQ1 is highly expressed in T-acute lymphoblastic leukemia (T-ALL) and promotes the development of T-ALL through promoting MYC RNA splicing^10^. Therefore, SHQ1 appears to participate in distinct activities depending on the cellular type and environment. There is need to understand the biological activities of SHQ1 in the context of cancer.

Some tumors grow due to endoplasmic reticulum (ER) stress, which is initiated by various drugs, oxidative stress, hypoxia, pH variation, and nutrient deprivation^11–13^. Unfolded protein response (UPR) is a well-characterized process that develops in cells in response to ER stress and relieves it^14^. UPR involves the activation of three distinct transmembrane proteins in the ER: activated transcription factor 6 (ATF6), PKR-like ER kinase (PERK) and inositol regulated endonuclease 1*α* (IRE1*α*)^15^. The three ER sensors interact with ER chaperone GRP78. Following ER stress, GRP78 dissociates from these sensors and triggers their activation^16^. ATF6 translocates to the Golgi and undergoes cleavage to convert to a 50-kDa protein p50ATF6, prior to its migration to the nucleus and activation of transcription^17^. PERK phosphorylates eIF2*α* to shut down protein translation, and activates the CHOP to upregulate the expressions of pro-apoptotic genes to initiate cell death^18^. CHOP induces the expression of GADD34 which promotes the dephosphorylation of eIF2*α* in a negative feedback loop to restore protein synthesis^19^. Apart from activating the extra nuclear splicing of the XBP1 mRNA (codes for a transcription factor), IRE1*α* induces a molecular chaperone p58^IPK^ which directly inhibits PERK activity to promote malignant progression^20^. The ER co-chaperone ERdj4 is induced by ER stress and selectively represses IRE1*α* signaling^21^. In addition, ATF6 could suppress IRE1*α* transcription^22^ and promote expressions of ERAD components including EDEM, HRD1, and Herp^15^, which causes degradation of IRE1*α*. Thus elevated ATF6 leads to restraint of IRE1*α*-XBP1 signaling^23^. Therefore, the UPR is a complex process with both positive and negative feedback loops.

In the present study, we demonstrated *SHQ1* is an ER-stress response gene that is transcriptionally regulated by p50ATF6 and XBP1s. SHQ1 binds to GRP78 and forms SHQ1/GRP78 to disrupt the interaction between GRP78 and ER sensors PERK/IRE1*α*/ATF6, which cause hyper-activation of UPR and apoptosis. We also show that SHQ1 promotes tumor apoptosis and sensitizes cells to chemotherapeutics. Taken together, our results demonstrate that SHQ1 is an ER stress response protein that positively regulates UPR in a feedback loop.

## Materials and methods

### Cell lines and culture

The HepG2 cells and the MDA-MB-231 cells were gifts from Dr. Zhigang Tian. HepG2 and HEK293T cells were cultured in complete DMEM supplemented with 10% NCS (GIBCO, 16010-159) at 37 °C and 5% CO_2_. MDA-MB-231 cells were cultured in complete DMEM supplemented with 10% FBS (Biological Industries, 04-007-1A) at 37 °C and 5% CO_2_. All cell lines tested negative for mycoplasma contamination, by PCR.

### Reagents and antibodies

The tissue microarray (HLivH160CS01) was purchased from SHANGHAI OUTDO BIOTECH Company. Tunicamycin (TM) (A61129) was purchased from Sangon Biotech. Thapsigargin (TG) (T9033) was purchased from Sigma. Puromycin was purchased from Biosharp (BS080A). Cisplatin (CDDP) (H20073653) was purchased from Qilu Pharmaceutical Company. Paclitaxel (PTX) (H20150351) was purchased from Hospira Australia Pty Ltd. Annexin V-FITC (640906) and PI (421301) were purchased from Biolegend. TUNEL Apoptosis Detection Kit (FA201-02) was purchased from TRANSGENE BIOTECH. NP-40 (9016-45-9) was purchased from Sangon Biotech. Hematoxylin (E607317), eosin (E607321), and neutral balsam (E675007) were purchased from Sangon Biotech. Anti-SHQ1 (SC-99629) was purchased from Santa Cruz. Anti-HA (66006-1-Ig), anti-GRP78 (11587-1-AP), anti-*β*-actin (60008-1-Ig), anti-*α*-Tubulin (66031-1-Ig), anti-GAPDH (60004-1-Ig) and anti-ATF6 (24169-1-AP) were purchased from Proteintech. Anti-Flag (F1804) was purchased from Sigma. Anti-PERK (#3192), anti-p-PERK (#3179S), anti-IRE1*α* (#3294), anti-cleaved caspase3 (#9664), and anti-cleaved PARP (#5625) were purchased from Cell Signaling Technology. Anti-p-IRE1*α* (ab48187) was purchased from Abcam. Anti-SHQ1 (IHC) (NBP1-92388) was purchased from Novus. Anti-Rabbit IgG-HRP (406401) and anti-Mouse IgG-HRP (405306) were purchased from Biolegend. Anti-PCNA (ZM-0213), horseradish peroxidase-conjugated goat anti-mouse/rabbit IgG (PV6000) and DAB (ZLI-9019) were purchased from ZSGB-BIO.

### Total protein extract

The cells or tissues were lysed using a NP-40 based buffer (Tris–HCl, pH 7.6, 50 mM; NaCl, 120 mM; EDTA, 1 mM; NP40, 1%) containing protease inhibitors cocktail (Sangon biotech, C600387) and incubated for 30 min at 4 °C with gentle mixing. Total protein concentration was determined using the Pierce BCA protein assay kit (Thermo Scientific, 23227) according to manufacturer’s protocol.

### Western Blot assay

An equivalent amount of total protein (30–50 μg) from each sample was separated on SDS–PAGE, proteins were transferred onto polyvinylidene difluoride membrane (Millipore, IPVH00010, 0.45 μm). The membranes were blocked in a solution containing 5% fat free milk (Biofroxx, 1172GR100) or 5% bovine albumin (Biofroxx, 4240GR500) at room temperature for 1 h, and then probed with the indicated primary antibodies at 4 °C overnight. Appropriate horseradish peroxidase-conjugated secondary antibodies were applied for 1 h at room temperature. Western bright ECL (Advansta, K-12045-D50) was used to detect immune-reactive proteins according to the manufacturer’s instructions.

### RNA extract and reverse transcriptase transcription PCR

Total RNA was extracted from tumor cells or tissues using Trizol reagent (ambion, 15596018) according to the manufacturer’s protocol, and cDNA was synthesized using M-MLV Reverse Transcriptase kit (Invitrogen, 28025021) and random primers (Sangon biotech, 100390265).

### qPCR

qPCR was performed with SYBR Green Mix (Vazyme, Q111-02) and relevant primers using an ABI-7300 real-time PCR machine. Relative mRNA expression was calculated from the comparative threshold cycle (Ct) values. Relative expression of the mRNA was calculated by 2^−ΔΔCt^ method and normalized to *GAPDH*. The primer for human *SHQ1*, Forward-AACTGCTCTTCTGGCACCAA, Reverse-GTGGCACTGCGGATTCAAAG, The primer for human *GRP78*, Forward-GTGGCCACTAATGGAGATACTCATC, Reverse–GCCCGTTTGGCCTTTTCTAC. The primer for human *CHOP*, Forward-CTGGAAGCCTGGTATGAGGAT, Reverse-CAGGGTCAAGAGTAGTGAAGGT, The primer for human *ERdj4*, Forward-GGAAGGAGGAGCGCTAGGTC, Reverse–ATCCTGCACCCTCCGACTAC. The primer for human *GADD34*, Forward-GCTTCTGGCAGACCGAAC, Reverse-GTAGCCTGATGGGGTGCTT. The primer for human *PDIA4*, Forward-AAGTCTCCCAGGGGCAGTTG, Reverse-GGGCGTACTTCAGCACGAAG. The primer for human *XBP1*, Forward-GTTAAGACAGCGCTTGGGGA, Reverse-TGCACGTAGTCTGAGTGCTG. The primer for human *XBP1s*, Forward-CTGAGTCCGCAGCAGGTGC, Reverse-TGGCAGGCTCTGGGGAAGG. The primer for human *ATF6*, Forward-CTTTTAGCCCGGGACTCTTT, Reverse-TCAGCAAAGAGAGCAGAATCC.

### Tumor xenografts

All animal experiments were approved by the Ethics Committee of the University of Science and Technology of China. Female BALB/c (nu/nu) mice (15–20 g, 4 weeks old) were purchased from the Model Animal Research Center of Nanjing University. HepG2 cells (5 × 10^6^/100 μl) stably expressing the control vector and shRNA of SHQ1 were injected subcutaneously into the right flank of female BALB/c nude mice (*n* = 6/group), respectively. Tumor volume (*V*) was estimated by measuring the longest diameter (*L*) and shortest diameter (*W*) of the tumor and calculated by formula *V* = (*LW*^2^)/2. For ER stress model in vivo, when the tumor volume was about 100 mm^3^, vehicle or TM 0.25 mg/kg was then injected weekly to the mice intraperitoneally for 4 weeks. Tumor size was measured every other day using a digital caliper. For tumor therapy studies, when the tumor volume was about 100 mm^3^, vehicle or PTX 15 mg/kg was then administered every second day to the mice intraperitoneally for a period of 18 days. Tumor size was measured every 2 days using a digital caliper. Mice were sacrificed at the end of the experiment. Tumors were harvested and weighed, and livers were separated for following study.

### Co-IP

Cells were washed twice by ice-cold PBS and trypsinized, and then lysed using a NP-40 lysis buffer (Tris–HCl, pH 7.6, 50 mM; NaCl, 120 mM; EDTA, 1 mM; NP40, 1%) containing with cocktail inhibitors for 30 min at 4 °C. Then the lysate was centrifuged at 12,000 × *g* for 10 min at 4 °C. Supernatant protein concentrations were determined as described above. An equivalent amount of proteins was incubated with anti-Flag or anti-HA antibody overnight, and then protein A/G beads were added to the mixture. 3 h later, immunoprecipitated proteins were washed three times with wash buffer (Tris–HCl, pH 7.6, 50 mM; NaCl, 120 mM; EDTA, 1 mM; NP40, 0.05%), the pellet (which contains bound proteins) was boiled in the SDS loading buffer and used for western blot analyses.

### GST pull down assay

GST-conjugated SHQ1, His-conjugated GRP78 proteins were expressed in BL21 *E. coli* and bound to the glutathione-sepharose beads (GE Healthcare, 28-9523-59) or His beads (GE Healthcare,29-0005-94) purified as described by the manufacturer. His-GRP78 proteins was incubated with GST or GST-SHQ1 proteins for 2 h at 4 °C in binding buffer (Tris–HCl, pH 8.0, 20 mM; NaCl, 200 mM; NP-40, 0.1%; *β*-ME, 0.3%), and then Glutathione-Sepharose beads were added to the mixture for 1 h at 4 °C. Immunoprecipitated proteins were washed three times with wash buffer (Tris–HCl, pH 8.0, 20 mM; NaCl, 200 mM; Triton X-100, 0.2%), the pellet was boiled in the SDS loading buffer for western blot analyses.

### Dual Luciferase Reporter assay

The SHQ1 promoter region (bp −307 to −107 upstream of the translation initiation site) was amplified by PCR. The primers for SHQ1 promoter, Forward-GGGGTACCCCAACCTCGAAAAAGGCTC, Reverse-GAAGATCTTCGGAGCTTCCGGCTCGAG. The primers for generating the point mutation on the promoter of human SHQ1 are: Forward- AGGCACCACTCGTTGTACCCTTTCTCCGCCCG, Reverse- CGGGCGGAGAAAGGGTACAACGAGTGGTGCCT. The resulting DNA fragments were constructed into pGL3-promoter luciferase reporter. HepG2 and MDA-MB-231 cells were transfected with SHQ1-luciferase constructs and Renilla luciferase plasmids (pRL-TK) which served as the internal control. After 12 h, cells were treated with TM for 12 h. Lysates were prepared using a Dual Luciferase reporter assay kit (Promega, E1960). The luciferase activities were determined using a Spectramax L Microplate Reader (Molecular Devices, San Jose, CA, USA). Firefly luciferase activities were normalized to that of Renilla luciferase in each case.

### HE staining

Part of liver and tumor tissues obtained from nude mice were fixed with 10% formalin and then embedded in paraffin. Then 5 μm sections were cut and stained with hematoxylin–eosin (HE). The stained sections were dehydrated in ethanol series, sealed with xylene and mounted with coverslip by adding neutral balsam (Sangon biotech, E675007-0100). The sections were photographed using a Zeiss Axioskop2 plus microscope (Carl Zeiss, Oberkochen, Germany) under bright field.

### Immunohistochemistry

Tumor/tissue sections were dewaxed in xylene and hydrated in an ethanol series. Antigen retrieval was performed by high pressure method for 2 min in buffer solution of different pH based on each antibody. After antigen retrieval, the sections were cooled naturally about 15 min, and then endogenous peroxidases were quenched using 3% hydrogen peroxide. The sections were washed by PBS three times. Subsequently, they were incubated overnight with anti-SHQ1, anti-GRP78, and anti-PCNA antibody at 4 °C, washed extensively and then incubated with horseradish peroxidase-conjugated goat anti-mouse/rabbit IgG at 37 °C for 30 min. The sections were visualized using diaminobenzidine (DAB). Finally, the sections were stained with hematoxylin, dried, and cover-slipped using neutral balsam.

### Annexin V-FITC/propidium iodide (PI) assay

Cells were seeded at a density of 2 × 10^5^ per well in six-well plates. After treatment with TM for indicated time, the cells were harvested with trypsin (without EDTA), washed with ice-cold PBS and binding buffer (HEPES, pH 7.4, 10 mM; NaCl, 140 mM; CaCl_2_, 2.5 mM) respectively. Then the cells were re-suspended in binding buffer, and incubated with FITC-conjugated anti-Annexin-V antibody in dark for 15 min. Before analyzing in a CytoFLEX flow cytometer (Beckman Coulter, Indianapolis, IN, USA), PI was added into each tube and incubated for 5 min. Data processing were analyzed by CytExpert software (Beckman Coulter, Indianapolis, IN, USA).

### TUNEL staining

Apoptosis in tumor tissue sections was detected using a TUNEL Apoptosis Detection Kit. Briefly, the sections were dewaxed in xylene, hydrated in a gradient ethanol series and then permeabilized with 0.1% Triton X-100 added in PBS at 37 °C for 8 min. After permeabilization, the sections were washed once with PBS. Sections were incubated with labeling solution containing terminal deoxynucleotidyl transferase (TdT) enzyme for 1 h at 37 °C. Then the sections were washed thrice with PBS. The sections were then incubated with Hochest 33342 (Dojindo, H342) at room temperature for 8 min and washed thrice with PBS. Finally, anti-fade mounting medium (Vector labs, H-1200) was added. Sections were examined with a microscope (NiKon (80i), Nikon (DS-Ril)) and NIS-Elements software.

### Real-time cell analysis (RTCA)

Cells were seeded at a density of 5000 per well in 16-well E-plates and incubated overnight. Then cells were treated with or without TM and chemotherapy drugs (PTX or CDDP), and placed in a RTCA (xCELLigence, Hangzhou, China) instrument for monitoring cell growth.

### MTT

The MDA-MB-231 breast carcinoma cell line was seeded in 96-well plates and incubated overnight. Cells were then treated with or without TM for 24, 72, 100, and 168 h. Cell proliferation was assessed by 5 mg/ml MTT (Sigma, M2128). The MTT solution was added to each well and incubated at 37 °C. 4 h later, the medium and MTT solution were removed, and DMSO was added to each well to dissolve the purple formazan at room temperature for 10 min. The absorbance was measured using the Biotek Epoch Multiskan spectrometer at 490 nm.

### Establishing SHQ1-knockdown HepG2 cell lines

pLKO.1-shRNA library purchased from Sigma-Aldrich LLC, Lentiviral vectors pLKO.1 or shRNA of SHQ1 were used for plasmid construction and transfected into HEK293T cells simultaneously with helper plasmids (VSVG:GAG:Rev:shRNA = 2:2:2:1). Lentiviral supernatants were collected 36–48 h post transfection. Approximately 2 × 10^5^ HepG2 cells in six-well plates were infected with lentiviral supernatants and 8 μg/ml polybrene (Sigma, H9268) for 24 h. And then the supernatants were replaced by the fresh medium with 2.5 μg/ml puromycin. Stable transfected cells were obtained by puromycin selection in 2–3 weeks. Finally, stably transfected cells were identified using western blot and quantitative real-time PCR analyses. ATF6-knockdown or XBP1-knockdown HepG2 cell lines were established in this same way.

### Establishing SHQ1-knockout MDA-MB-231 cell lines

The target sequence of SHQ1 guide RNA-2 is TACGATCAGGAGTGTTGCAACGG, the target sequence of SHQ1 guide RNA-5 is GAAGTCCGGATCCTGGCTG. LentiCRISPR vectors and SHQ1 gRNA were used for plasmid construction and transfected into HEK293T cells simultaneously with helper plasmids (PMD2.G:psPAX5:lentiCRISPR = 3:1:4). Lentiviral supernatants were generally collected 24–36 h post transfection. Approximately 2 × 10^5^ MDA-MB-231 cells in six-well plates were infected with lentiviral supernatants and 8 μg/ml polybrene for 24 h. And then the supernatants were replaced by the fresh medium with 2 μg/ml puromycin. Single transfected cells were sorted into individual wells by limiting dilution analysis (LDA) in 96-well plates, expanded into cell lines, and screened by western blot.

### Library preparation and RNA sequencing

Total RNA was prepared from MDA-MB-231 cells after treating with or without TM for 20 h and subjected to RNA-seq (Novogene, Beijing, China). Sequencing libraries were generated using NEBNext Ultra^TM^ RNA Library Prep Kit for Illumina (NEB, Ipswich, MA, USA). The clustering of the index-coded samples was performed on a cBot Cluster Generation System according to the manufacturer’s instructions. After cluster generation, the library preparations were sequenced on an Illumina Hiseq platform and 125 bp/150 bp paired-end reads were generated.

### GO-enrichment analysis of differentially expressed genes

Differential expression analysis was performed between parent and SHQ1-knockout MDA-MB-231 cells and cells was performed using the DESeq2 R package (1.16.1). Gene ontology (GO) enrichment analysis of differentially expressed genes was implemented by the cluster Profiler R package. GO terms with corrected *p* value < 0.05 was considered significantly enriched by differential expressed genes.

### Subcellular structure protein extraction

The separation and preparation of cytoplasmic, membrane and nuclear protein extracts from HepG2 cells were performed by Subcellular Structure Protein Extraction Kit (Sangon biotech, C500073) according to the manufacturer’s instructions. The cytoplasm, membrane, and nuclear protein were extracted in sequence. The distribution of proteins in each fraction was analyzed by western blot.

### Statistical analysis

Sample size were similar to or larger than those reported in previous published studies. There are no samples or animals excluded in our analysis. *p* values in this study were calculated by two-tailed unpaired Student’s *t* test and two-way ANOVA analysis using GraphPad Prism 6.0 (GraphPad Software). The significance level for the statistical analysis was set at *p* < 0.05. Data are shown as mean value ± SD of at least three independent experiments.

## Results

### SHQ1 is an ER stress response gene

**Fig. 1:**
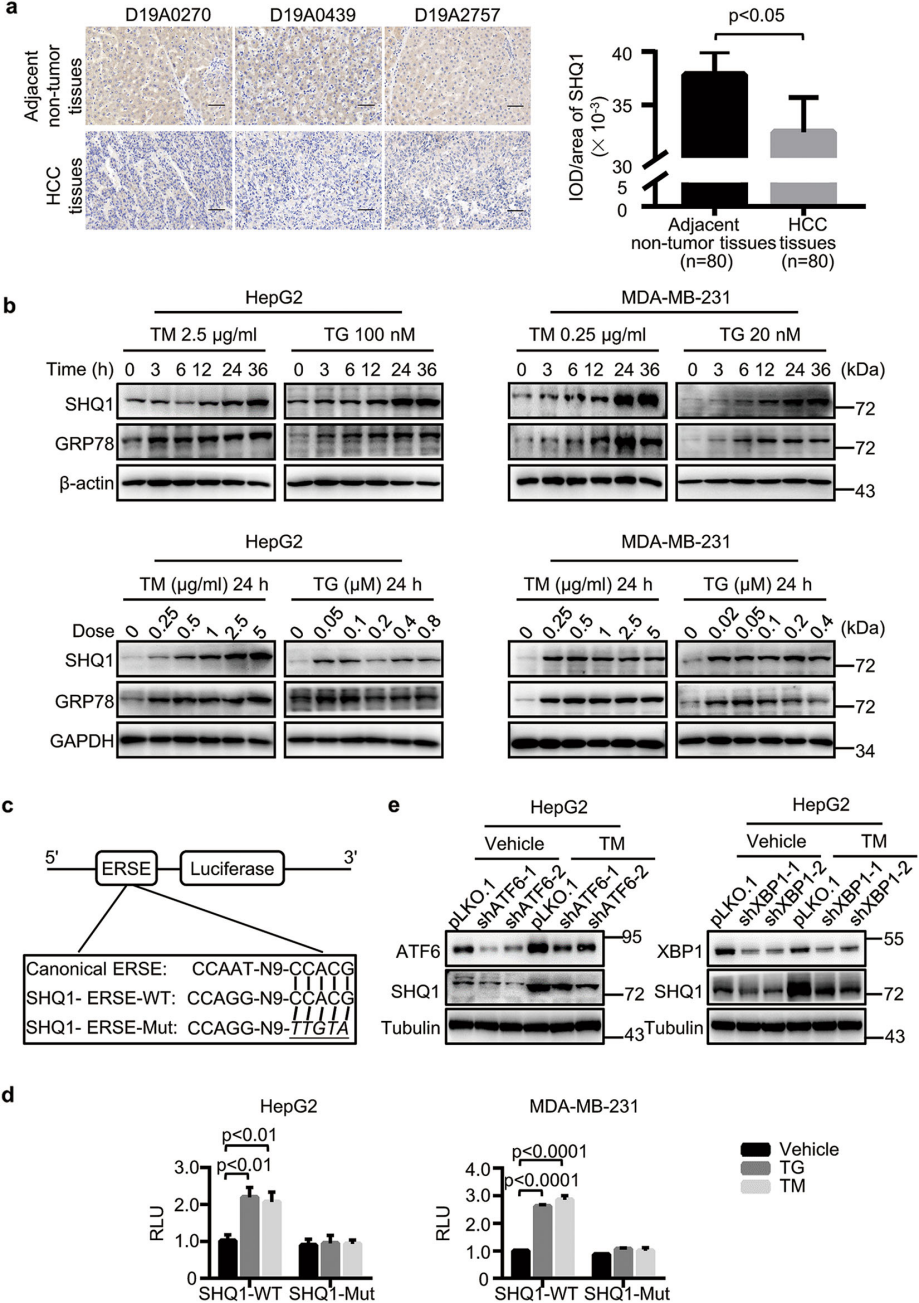
SHQ1 is transcriptionally upregulated during ER stress. **a** The tissue microarray containing 80 paired tumor tissues and adjacent non-tumor tissues from primary hepatocellular carcinoma (HCC) patients were used for determining the expression of SHQ1 by immunochemistry (*n* = 80). Three representative sample pairs are shown (200×). All scale bars indicate 100 μm. The integral optical density (IOD) per area of SHQ1-positive staining was analyzed using IMAGE-PRO PLUS software and the average IOD/area from all samples is presented in the graph. **b** HepG2 and MDA-MB-231 cells were treated with indicated doses of ER-stress inducers TM or TG over a 36 h time course or various doses of the TM or TG for 24 h. SHQ1 and GRP78 accumulation were analyzed via immunoblotting with the indicated specific antibodies. **c** Diagram of luciferase reporters containing either ERSE-WT sequence located at the *SHQ1* promoter or ERSE-MUT lacking ability to respond to ER stress. The mutated nucleotides were indicated as italic and underlines. WT wild type. Mut mutated. **d** Cells were transfected with either SHQ1-WT or SHQ1-Mut ERSE reporters and cultured for 12 h, then these cells were treated with TM or TG for 12 h. The cell lysates were then assayed for dual luciferase activity using a commercially available kit. RLU relative luciferase units. **e** ATF6-KD (shATF6-1, shATF6-2) HepG2 cells or XBP1-KD (shXBP1-1, shXBP1-2) HepG2 cells and control HepG2 cells (pLKO.1) were treated with 2.5 μg/ml TM as indicated for 24 h. The protein levels of SHQ1, ATF6, and XBP1 were examined by western blotting. Data shown in this figure are representative of at least three independent experiments. Quantified data represent the mean ± SD, two-tailed unpaired Student’s *t* test.

Previous studies have associated the lack of the chromosome 3p SHQ1 locus with both prostate and cervical carcinoma^4,5^, and SHQ1 expression is dramatically reduced in human primary prostate tumors^3^, findings suggesting that SHQ1 may limit the formation of such tumors. We next determined if the expression of SHQ1 is somehow altered in a tissue microarray comprising 80 primary hepatocellular carcinomas (HCC). The expression of SHQ1 was lower in HCC tumor tissues than that in adjacent non-tumor tissues (Fig. 1a), suggesting tumors tend to lose SHQ1 for promoting their growth. Some studies have indicated that UPR activated by ER stress is involved in the initiation and progression of HCC^13^, and UPR signaling promotes a malignant phenotype and helps tumors become more resistant to chemotherapy in breast cancer^24^. Deletion of *FOXP1-SHQ1* region is seen in 27% of breast cancer^25^. To investigate the biological functions of SHQ1 in tumors, we treated HepG2 (HCC) and MDA-MB-231 (metastatic breast carcinoma) cell lines with two classic ER stress inducers: TM and Thapsigargin (TG). Attesting to the successful induction of ER stress, we detected TM-induced and TG-induced increases in the accumulation of the well-known ER stress marker protein GRP78 (Fig. 1b). SHQ1 protein expression was similarly induced by TM and by TG, and this induction was both dose-dependent and time-dependent manner (Fig. 1b). A sequence CCACG similar to the well-known ER-stress response element (ERSE) was present in the *SHQ1* promoter^26^, so we constructed luciferase reporters driven by either the wild type (WT) promoter or a mutated version with each base substitution in the sequence CCACG of ERSE motifs which is similar to a previously reported mutated ERSE^27,28^ (Fig. 1c). TM and TG treatment of HepG2 and MDA-MB-231 cells harboring these constructs revealed luciferase reporter expression as driven by the WT-ESRE but not the mutated sequence (Fig. 1d), indicating that the ERSE-like element in the *SHQ1* promoter functionally contributes to the observed increased SHQ1 accumulation upon treatment with ER stress-inducers. Previous studies have demonstrated that the transcription factors ATF6 and XBP1 activate transcription of ER stress response genes^27,29,30^, and we observed that both protein and RNA levels of SHQ1 were significantly decreased in whether ATF6-knockdown (ATF6-KD) HepG2 cells or XBP1-knockdown (XBP1-KD) HepG2 cells in ER stress (Fig. 1e and Supplementary Fig. 1a–c). Furthermore, we found that over-expression of ATF6 and of XBP1 significantly increased the SHQ1 levels in HEK293T cells (Supplementary Fig. 1d). Taken together, our results indicated SHQ1 is an ER-stress response gene.

### SHQ1 promotes UPR-mediated cell growth suppression

**Fig. 2:**
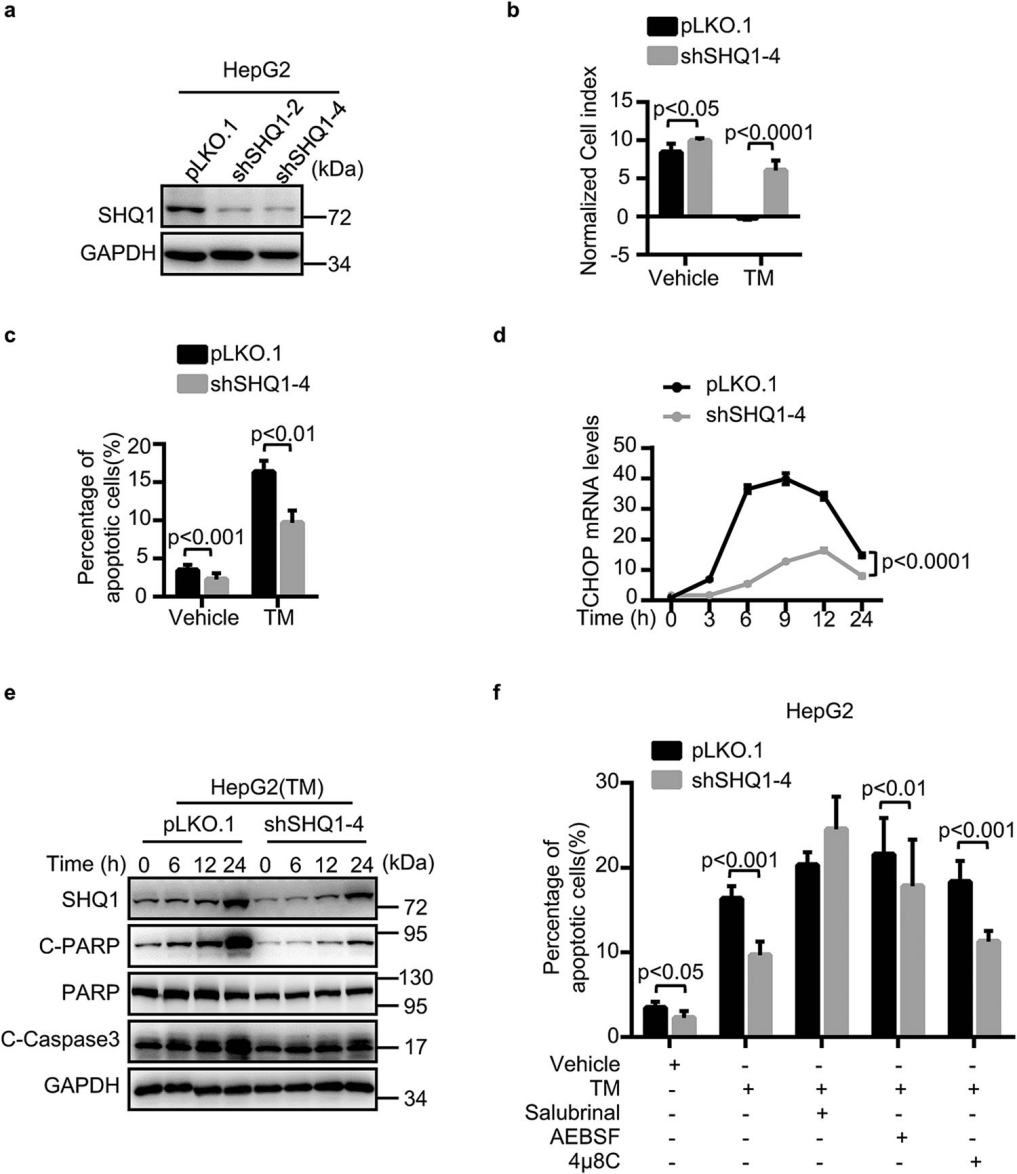
SHQ1 promotes apoptosis in HepG2 cancer cells. **a** Establishment of SHQ1-knockdown (SHQ1-KD) and control cells using RNA interference technology with two independent shRNAs. Western blotting was used to confirm SHQ1 protein deficiency in the shRNA-knockdown HepG2 cells. **b** SHQ1-KD HepG2 cells and control HepG2 cells were either treated with 0.25 μg/ml TM as indicated, the cell growth were monitored by real-time cell analysis. The bar graph shows the normalized cell index at 100 h in each group. **c** The proportion of cells exhibiting apoptosis was measured using Annexin V/PI staining by flow cytometry. **d** The mRNA levels of CHOP were determined by qPCR at the indicated time points. **e** The changes in apoptotic proteins were examined by western blotting at the indicated time points. C-PARP cleaved PARP. C-Caspase 3 cleaved caspase 3. **f** SHQ1-KD HepG2 cells and control HepG2 cells were pre-treated with 50 μM of Salubrinal (a PERK inhibitor), 50 μM of 4*μ*8C (an IRE1*α* inhibitor), and 150 μM of AEBSF (an ATF6 inhibitor) for 1 h before incubating with 2.5 μg/ml TM for 48 h. The proportion of cells exhibiting apoptosis was measured using Annexin V/PI staining by flow cytometry. Data shown in this figure are representative of three independent experiments. Quantified data represent the mean ± SD, data shown in **b**, **c**, **f** were analyzed by two-tailed unpaired Student’s *t* test, and the data shown in **d** were analyzed by two-way ANOVA analysis.

To address the potential ER-stress-related function of SHQ1, we generated shRNA-mediated SHQ1 knockdown (SHQ1-KD) HepG2 cells and CRISPR/Cas9 gene-edited SHQ1 knockout (SHQ1-KO) MDA-MB-231 cells (Fig. 2a and Supplementary Fig. 2a). We explored the growth phenotypes in SHQ1-deficient cells and control cells. Interestingly, our RTCA-based or MTT assessment of cell growth showed that SHQ1 deficiency conferred a growth advantage to both TM-treated HepG2 and TM-treated MDA-MB-231 cells (Fig. 2b and Supplementary Fig. 2b). Given that the induction of ER stress is a classic inducer of intrinsic apoptosis^31,32^, we next used flow cytometry to determine the extent of apoptosis in the control and SHQ1-deficient TM-treated tumor cells. We observed a significant decline in apoptosis in SHQ1-deficient HepG2 and MDA-MB-231 cells (Fig. 2c and Supplementary Fig. 2c). Thus, SHQ1 promotes apoptosis in highly ER-stressed tumors. Moreover, the levels of apoptosis markers including CHOP, the cleaved forms of caspase3 and PARP, were significantly decreased in TM-treated SHQ1-deficient cells compared to TM-treated control tumor cells (Fig. 2d, e and Supplementary Fig. 2d, e). There are several UPR pathways, so we treated HepG2 cells with the inhibitors Salubrinal, AEBSF, and 4μ8c to, respectively, suppress activation of the known ER sensors (PERK, ATF6, and IRE1*α*) to explore which pathway(s) function in the observed apoptosis in SHQ1-KD HepG2 cells. Treatment with the PERK inhibitor Salubrinal, restored apoptosis in SHQ1-deficient cells (Fig. 2f). Taken together, SHQ1 promotes ER-stress-induced tumor apoptosis via PERK-signaling pathway.

**Fig. 3:**
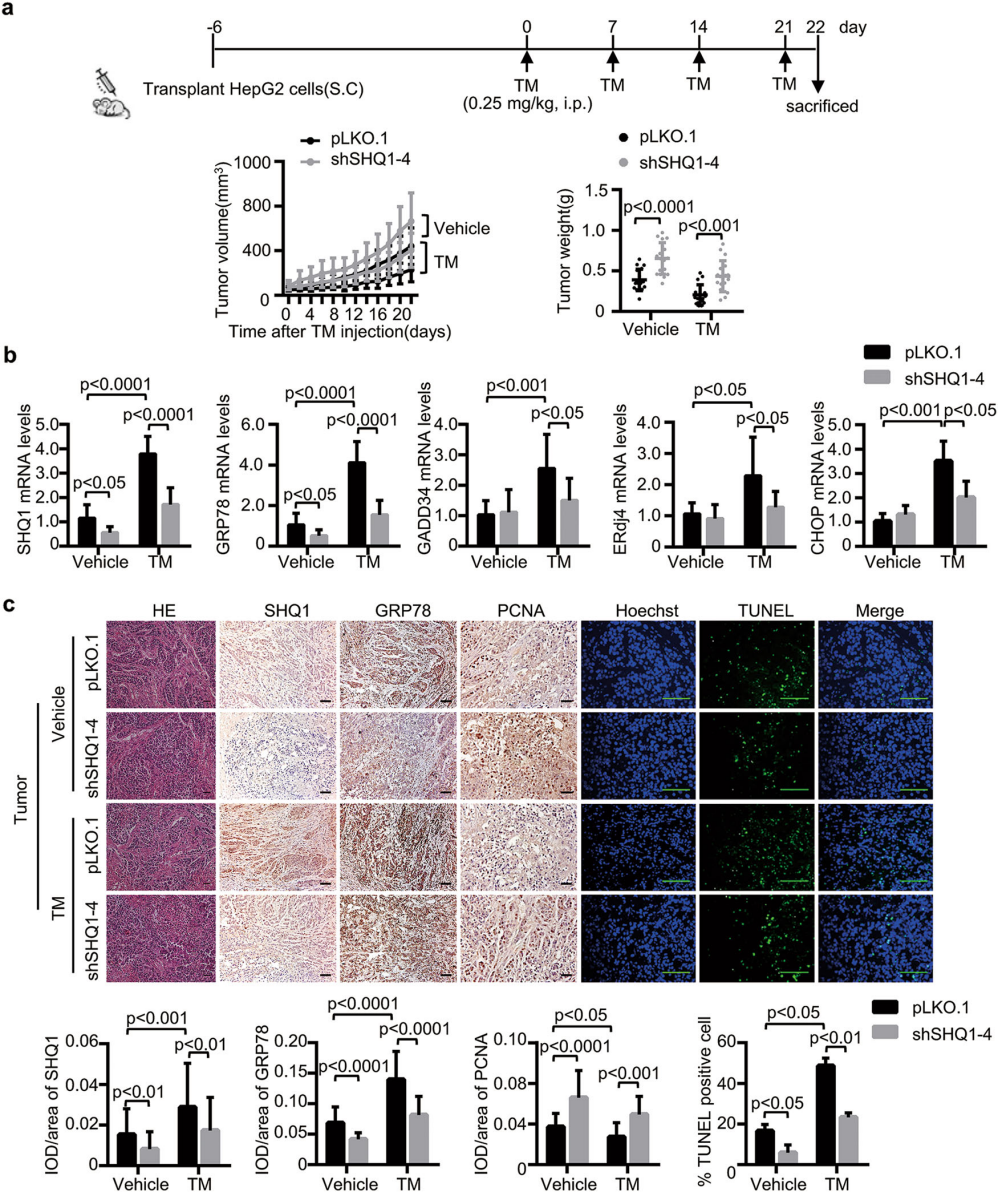
SHQ1 inhibits tumor growth in xenograft model upon ER stress. **a** Scheme of treatment in a HepG2 xenograft tumor model with ongoing induced ER stress. Tumor volumes were measured every other day and plotted. The dot graph on the right shows dissected tumor weights at the end of experiment. Each dot represents the tumor mass from one mouse (*n* = 18). **b** UPR-related gene expression in the tumor xenografts was measured using qPCR (*n* = 3 mice for each group). **c** The expression of SHQ1, GRP78, and PCNA in the tumor tissues was analyzed using immunohistochemistry and presented as the IDO/area ratio (*n* = 3 mice for each group). Representative images are shown (×200). Apoptosis was detected using in situ TUNEL staining. The percentage of TUNEL-positive cells/total cells was analyzed in each field under fluorescent microscopy (×400). At least 10 independent random fields were counted for quantifying the data. All scale bars indicate 100 μm. Data shown in this figure are representative of three independent experiments. Quantified data represent the mean ± SD, two-tailed unpaired Student’s *t* test.

Based on our in vitro results, we next examined if the loss of SHQ1 promotes tumor growth in vivo. An ongoing ER stress model in HepG2 xenograft tumors were established by subcutaneously transplanting SHQ1-KD HepG2 cells and control HepG2 cells into nude mice and followed by intraperitoneal injection of TM once a week (Fig. 3a). The success of this model was confirmed by a significantly increased expression of representative UPR-related genes in the xenograft tumors after TM injection (Fig. 3b). SHQ1 deficiency was sufficient to accelerate tumor growth after injection with TM (Fig. 3a). Consistently, loss of SHQ1 resulted in a significant increase in tumor weight (Fig. 3a). In addition, the mRNA levels of UPR-related genes in SHQ1-KD HepG2 xenograft tissues were significantly lower than that in control group (Fig. 3b). These results suggested that SHQ1 affected UPR in tumors. Immunohistochemical analyses revealed that the expression of GRP78 was significantly decreased in SHQ1-KD tumors (Fig. 3c).Tumor xenografts were stained with a PCNA antibody for detecting proliferation. A relatively increased PCNA immunoreactivity was found in the SHQ1-deficient group, when compared to the control (Fig. 3c). We have also determined apoptosis in HepG2 xenografts by using TUNEL assays. Treatment with TM caused a decreased apoptosis in tumor tissues lacking the SHQ1 (Fig. 3c). Altogether, these results revealed SHQ1 inhibited tumor growth upon ER stress.

### SHQ1 competitively binds with GRP78 from ER sensors and induces hyper-activation of UPR

**Fig. 4:**
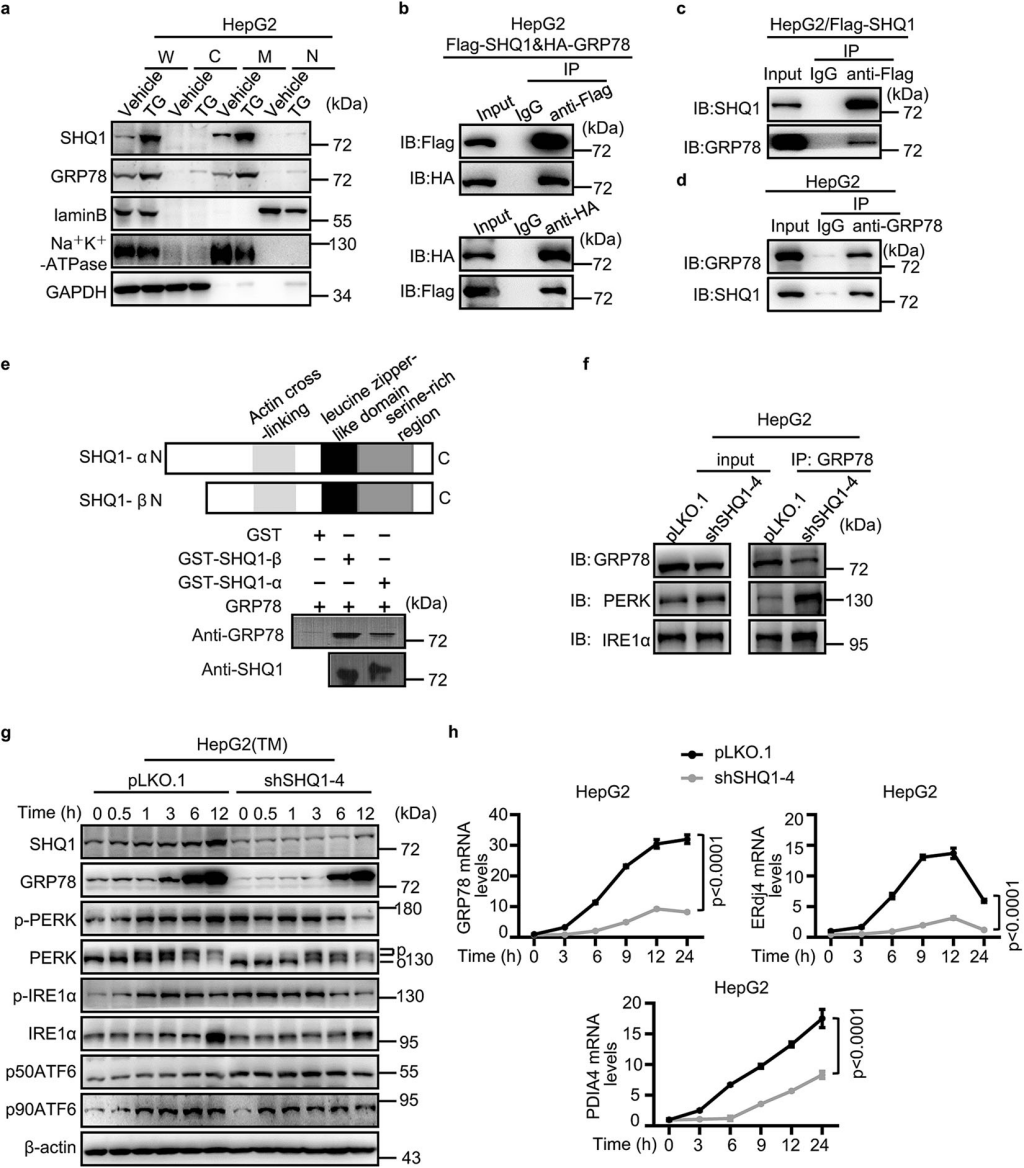
SHQ1 interacts with GRP78 and hyper-activates UPR. **a** HepG2 cells were treated with 100 nM TG as indicated. A commercially available Subcellular Structure Protein Extraction assay kit was used for fractionating the subcellular components. Abbreviations: W whole cell lysate, C cytosolic fraction, M membrane fraction, N nuclear fraction. **b**, **c** HepG2 cells were transfected with either Flag-SHQ1 vectors alone or in combination with HA-GRP78 vectors, and were cultured for 24 h. Co-immunoprecipitation analysis for determining the relationship between GRP78 and SHQ1. Anti-Flag or anti-HA antibody was used for immunoprecipitation. The immunorecipitates were determined using western blotting with indicated specific antibodies. **d** Co-immunoprecipitation analysis for determining the relationship between GRP78 and SHQ1. Anti-GRP78 antibody was used for immunoprecipitation. The immunorecipitates were determined using western blotting with indicated specific antibodies. **e** N-terminal GST fusion vectors expressing the full length SHQ1 (SHQ1-*α*) or a naturally expressing truncated version lacking the N-terminus (SHQ1-β) were constructed. Purified recombinant proteins (2 μg) were co-incubated for 2 h in a binding buffer at 4 °C. The immunoprecipitated proteins were detected by western blotting with indicated specific antibodies. **f** Co-immunoprecipitation analysis for determining the relationship between GRP78 and ER sensors in SHQ1-KD and control HepG2 cells. Anti-GRP78 antibody was used for immunoprecipitation. The amounts of GRP78, PERK, and IRE1*α* present in the immunoprecipitates were measured using western blotting analyses with the indicated specific antibodies. **g** SHQ1-KD HepG2 cells and control HepG2 cells were treated with TM for the indicated time points, and the expression of ER sensors was monitored using western blotting. O and P indicate mobility changes for non-activated PERK and activated PERK by phosphorylation. p-PERK, phosphorylated PERK; p-IRE1*α*, phosphorylated IRE1*α*; p50ATF6, activated form of ATF6; p90ATF6, the full length precursor form of ATF6. **h** SHQ1-KD HepG2 cells and control cells were treated with TM for the indicated time points, and qPCR was performed to evaluate the expression of UPR-related genes (*n* = 3). Quantified data represent the mean ± SD, two-way ANOVA analysis. Data shown in this figure are representative of three independent experiments.

Since SHQ1 upregulated UPR-related genes, we supposed SHQ1 may act upstream of UPR. So we speculated that SHQ1 may interact with GRP78, a major chaperone of UPR. We first analyzed the subcellular location of SHQ1 and GRP78 in HepG2 cells by employing subcellular fractionation assays. The results showed SHQ1 and GRP78 both mainly located in membrane fraction and their accumulations in membrane fraction were significantly increased after TG treatment (Fig. 4a), providing spatial possibilities for their interactions. Indeed, co-immunoprecipitation analyses showed that HA-tagged GRP78 could be co-immunoprecipitated by Flag-tagged SHQ1, and Flag-tagged SHQ1 was co-immunoprecipitated by HA-tagged GRP78 in HepG2 cells transfected with the expression vectors for these proteins (Fig. 4b). Additionally, HepG2 cells were immunoprecipitated with an anti-Flag antibody (SHQ1) and immunoprecipitates were blotted with an anti-GRP78 antibody, the results showed SHQ1 interacts with endogenous GRP78 (Fig. 4c). In order to verify their endogenous interaction, an anti-GRP78 antibody was used to immunoprecipitate in HepG2 cells and immunoprecipitates were blotted with an anti-SHQ1 antibody. The results showed that endogenous SHQ1 could be co-immunoprecipitated by endogenous GRP78 (Fig. 4d). To confirm if SHQ1 directly interacted with GRP78, a glutathione S transferase (GST) pull-down assay was performed. Full length SHQ1 (α) and naturally occurring variant lacking the N-terminus (β) were expressed as GST fusion proteins. Purified recombinant proteins were incubated together and proteins were pulled down using GST-agarose beads. Both the α and β forms of SHQ1 were able to bring down GRP78. GST alone did not bind to GRP78. These results demonstrate that SHQ1 and GRP78 directly interact with each other, and establish that the N-terminus of SHQ1 is dispensable for such an interaction (Fig. 4e).

GRP78 is a master regulator in the ER that associates with ER sensors^33,34^. We hypothesized that SHQ1 may alter the interaction(s) between GRP78 and ER sensor. The interactions between GRP78 and ER sensors in SHQ1-KD HepG2 cells and control HepG2 cells were examined using co-immunoprecipitation analyses. Consistent with our speculation, these studies revealed that depletion of SHQ1 significantly enhanced the interaction of GRP78 and PERK (Fig. 4f). The interaction between GRP78 and IRE1*α* was slightly enhanced by the loss of SHQ1 (Fig. 4f). Thus SHQ1 appears to negatively regulate the interactions between GRP78 and ER sensors.

Next, we investigated the effects of SHQ1 on the activation of ER sensors. We treated HepG2 and MDA-MB-231 cells with or without TM and determined the expression of GRP78 and ER sensors (PERK, IRE1*α*, and ATF6). The steady-state levels of GRP78 and ER sensors were not significantly affected. However, while there was a time-dependent increase in these sensors in the control cells, deletion or depletion of SHQ1 in HepG2 and MDA-MB-231 caused a reduced expression of GRP78 and ER sensors (Fig. 4g and Supplementary Fig. 3b). The levels of phosphorylated PERK increased gradually after TM treatment in control HepG2 cells, while its activation in SHQ1-KD HepG2 cells was slightly impaired (Fig. 4g). The phosphorylated IRE1*α* levels reached a peak at 24 h after TM treatment in control MDA-MB-231 cells, however, its levels reached a peak at 6 h and then started to decline with the loss of SHQ1 (Supplementary Fig. 3b), indicating that SHQ1 caused an extended period of UPR activation. These results suggested SHQ1 may hyper-activate UPR. The RNA-seq analysis showed genes related to ER and ER to Golgi transport were significantly downregulated in SHQ1-KO MDA-MB-231 cells when compared to control cells (Supplementary Fig. 3a). In addition, we observed that the mRNA levels of UPR-related genes such as *GRP78*, *ERdj4*, and *PDIA4* were significantly lower in TM-treated SHQ1-deficient cells (Fig. 4h and Supplementary Fig. 3c). Together, these results demonstrated SHQ1 binds with GRP78 to disrupt its interaction with ER sensors, leading to hyper-activation of UPR.

### SHQ1 improves chemotherapy sensitivity

**Fig. 5:**
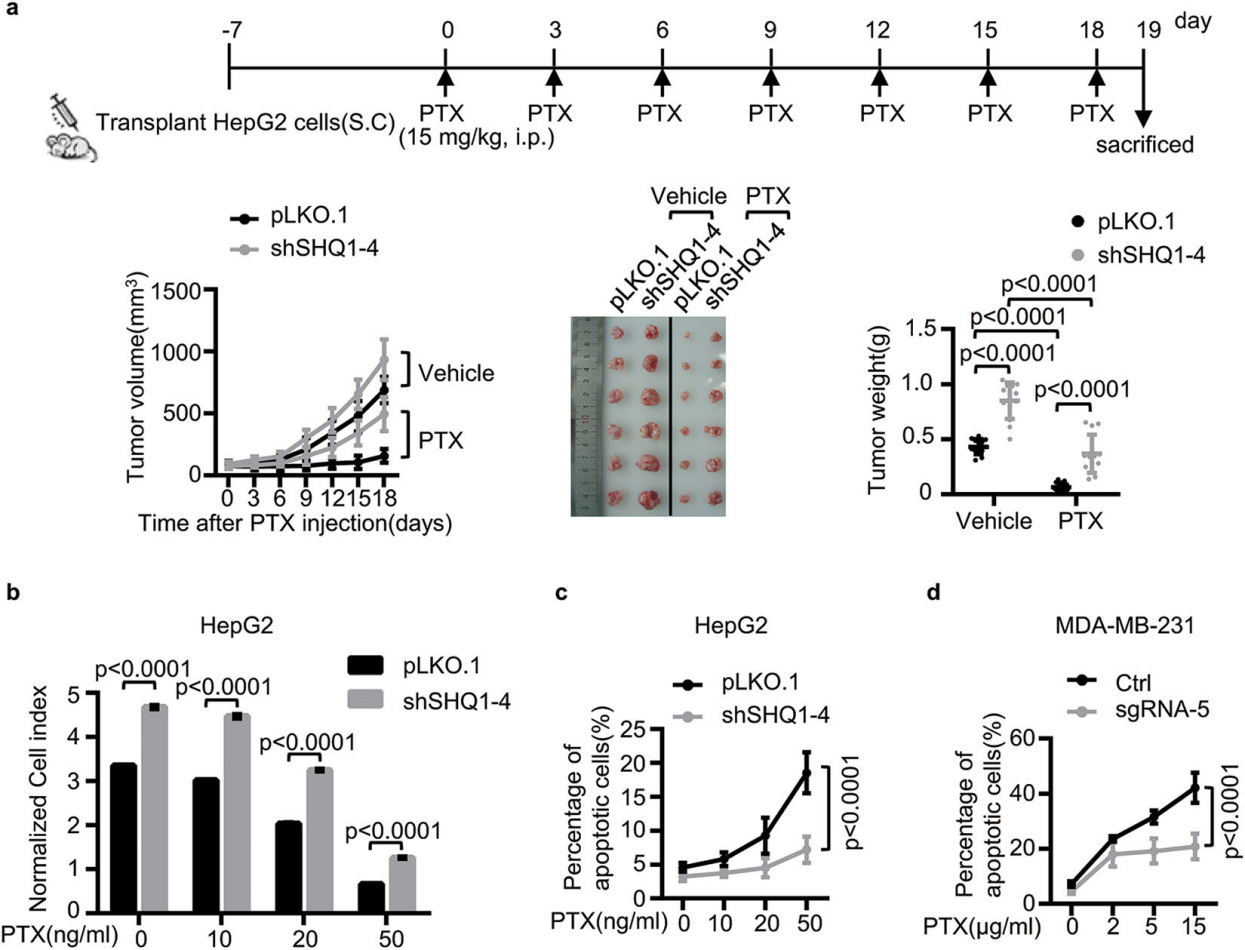
SHQ1 sensitizes cells to chemotherapy drug PTX in vitro and in vivo. **a** A scheme for the treatment of xenograft tumors with PTX. Tumor volumes were measured every 2 days and plotted. The dissected tumor images and tumor weights (dot plot) are presented (*n* = 13). **b**, **c** SHQ1-KD HepG2 cells and control cells were treated with different doses of PTX as indicated. **b** Cell growth was assessed via real-time cell analysis. The bar graph shows the normalized cell index at 100 h in each group. **c** The proportion of cells exhibiting apoptosis was measured using Annexin V/PI staining by Flow cytometry after treating them with PTX for 48 h. **d** SHQ1-knockout (SHQ1-KO) MDA-MB-231 cells were established by using guide RNA (sgRNA-5) through CRISPR/Cas9 technology. The SHQ1-KO (sgRNA-5) MDA-MB-231 cells and control (Ctrl) MDA-MB-231 cells were treated with different doses of PTX for 48 h as indicated. The proportion of cells exhibiting apoptosis was measured using Annexin V/PI staining by flow cytometry. Data shown in this figure are representative of three independent experiments. Quantified data represent the mean ± SD, two-tailed unpaired Student’s *t* test.

Insensitivity to anti-cancer drug-induced cell death is one of the mechanisms which could be regulated by UPR^35^. Based on our above results, we hypothesized that SHQ1 may suppress cancer drug resistance by promoting UPR. To test this, we evaluated the impact of paclitaxel (PTX) on HepG2 xenograft tumor growth. We subcutaneously transplanted control HepG2 cells and SHQ1-KD HepG2 cells into nude mice. Once palpable tumors developed, we treated these mice with paclitaxel (PTX) every 2 days (Fig. 5a). In the control tumors, PTX strongly retarded tumor growth (Fig. 5a). The SHQ1-KD tumor xenografts were significantly more resistant to PTX than the controls, as determined by increased tumor volume and tumor weight in this group (Fig. 5a). Thus, SHQ1 appears to sensitize cells to chemotherapeutics. Furthermore, SHQ1-KD HepG2 cells and control HepG2 cells were treated with different doses of PTX. RTCA-based assessment of cell growth showed SHQ1 deficiency conferred a growth advantage. PTX could not inhibit cell growth efficiently in the absence of SHQ1 (Fig. 5b), and the percentage of apoptotic cells in SHQ1-KD HepG2 cells were significantly lesser than those in similarly treated control HepG2 cells (Fig. 5c). A similar effect of SHQ1 on PTX-sensitivity of MDA-MB-231 was noted (Fig. 5d). To confirm the role of SHQ1 in drug sensitivity, we treated SHQ1-KO MDA-MB-231 cells and control MDA-MB-231 cells with another chemotherapy drug cisplatin (CDDP). Cell growth and apoptotic assays in MDA-MB-231 cells showed that SHQ1 was required for suppressing growth in response to CDDP (Supplementary Fig. 4a, b). Taken together, these studies demonstrate that presence of SHQ1 enhances tumor sensitivity via facilitating to chemotherapy.

## Discussion

In this study, we demonstrated a novel role for SHQ1, a protein thought to control the level of small RNAs of the Sno and Sca family, in positively regulating UPR.

GRP78 is a master regulator of UPR. During ER stress, it dissociates from the ER-stress sensors and binds to unfolded or misfolded proteins to help cells to resolve ER stress^16^. GRP78 is highly expressed in some malignant tumors and patients with high tumor-GRP78 levels have a poor prognosis^36–38^. The up-regulation of GRP78 promotes ER homeostasis and allows cells to survive^16,33^. Recent studies have indicated a lead compound HA15 interacts with GRP78 and specifically inhibits the GRP78 activity, inducing ER stress and leading to cancer cell death^39^. However, in our study, we found SHQ1 is positively correlated with GRP78, which seems contradict the observation that SHQ1-deficient cells have a better growth advantage. We interpret that availability of SHQ1 competitively sequesters GRP78, and prevents its association with ER stress sensors, thereby hyper-activating UPR. A consequence of persistent UPR activation is apoptosis^18^. Consistent with our results, previous studies have demonstrated transient activation of GRP78 promotes cell survival by ER stress, while persistent GRP78 activation would lead to cell apoptosis via PERK/eIF2*α*/CHOP pathway^40^. Interestingly, it has been reported GRP78 interacts with caspase7 and prevents the cleavage of caspase 7, thus protecting cancer cells from apoptosis^41^. We speculated the formation of GRP78/SHQ1 complex may also block the inhibitory effects of GRP78 on caspase7, for promoting apoptosis. It is likely the effect of GRP78 on cancer development is not unequivocal. GRP78 may regulate these diverse effects depending on the cellular environment and its interacting partners. In addition, we herein showed SHQ1 directly interacted with GRP78, and N-terminal (CS) domain of SHQ1 is dispensable for their interaction. Consistently, previous studies have reported loss of Shq1p inhibits cell viability in yeast^3^, and C-terminal region of shq1p is different from human SHQ1 of which the C-terminal is rich in serine/threonine that may be essential for its anti-cellular effect^3^. Thus, we speculate that the C-terminus of SHQ1 is essential for the interaction with GRP78. Previous studies have indicated that the C-terminal sequence Lys-Asp-Glu-Leu (KDEL) of GRP78 helps it mainly localize in ER membrane^34^. Our results showed SHQ1 and GRP78 both localized in membrane and interacted with each other. Although the KDEL sequence is not presented in SHQ1 protein, it is reasonable to speculate that the localization to ER of SHQ1 might be caused by the recruitment of GRP78. Our results showed SHQ1 knockdown led to decrease in ER stress response genes such as GRP78. The separate mechanisms may contribute to the observations in this study. GRP78 usually goes through ubiquitin-mediated proteasome degradation pathway by the E3 ligase GP78^42^. GRP78 levels were concurrently decreased in SHQ1-deficient cells when treated with TM, suggesting that SHQ1 may prevent GRP78 degradation by interacting with GRP78. On the other hand, SHQ1 binds to GRP78 to release ER sensors PERK/IRE1*α*/ATF6 and causes hyper-activation of UPR. Thus, SHQ1 deficiency inhibits activation of UPR, leading to decrease in transcription of ER stress response genes.

Many cancer cells are accompanied with constitutive UPR because of the microenvironment of solid tumors, and UPR initially protects cells from hostile microenvironment^12^. Under prolonged and serve ER stress, UPR promotes apoptosis^11^. UPR acts as a double-edged sword during ER stress-mediated apoptosis and plays a role in resistance to chemotherapy^35^. Resistance to chemotherapy is common in many solid tumors^35^. UPR initially is cytoprotective for tumors to adapt to cellular stress and may confer resistance to chemotherapeutics, thus leading to low therapeutic efficacy^43^. However, some studies have indicated that hyper-activation of UPR may improve chemotherapy. It has been reported that targeting UPR regulators may eradicate doxorubicin-resistant breast cancer cells^44^. The compound of thiazole benzenesulfonamides overcomes BRAF inhibitor resistance in melanoma via triggering ER stress-induced cell death^39^. In our study, we showed SHQ1 increased the sensitivity of tumors to paclitaxel (PTX) or cisplatin (CDDP) via hyper-activating UPR, suggesting that induction of SHQ1 expression might provide a therapeutic opportunity. Some studies have reported that TG increased 10–12 fold more apoptosis when combined with PTX in prostate cancer^45^. Furthermore, combinations of TM with CDDP suppressed xenograft tumor growth more efficiently than TM or CDDP alone did^46^. Based on our results, Combination of SHQ1 with chemotherapy drugs may be a new strategy to improve cancer therapy.

SHQ1 positively regulates UPR for promoting tumor-suppressive effects in a variety of solid tumor cells. Consistent with previous studies that SHQ1 is lost in primary prostate cancer^3^, SHQ1 expression is also significantly downregulated in the primary HCC tumor tissues compared to adjacent non-tumor tissues, suggesting its tumor-suppressive property. Alteration frequency of SHQ1 gene including mutation and deletion is <5% which is relatively low in a wide of tumors from TCGA database. Thus SHQ1 expression appears to be downregulated for promoting solid tumor growth. Indeed, tumor growth, and resistance to drug-induced apoptosis are enhanced in HepG2 xenograft tumors lacking SHQ1. However, SHQ1 is highly expressed in T-ALL and promotes the development of T-ALL^10^, which is contrary to previous reports of prostate cancer and cervical cancer. The relevance of high and the mechanisms of SHQ1 in hematologic tumors need to be studied more thoroughly.

**Fig. 6:**
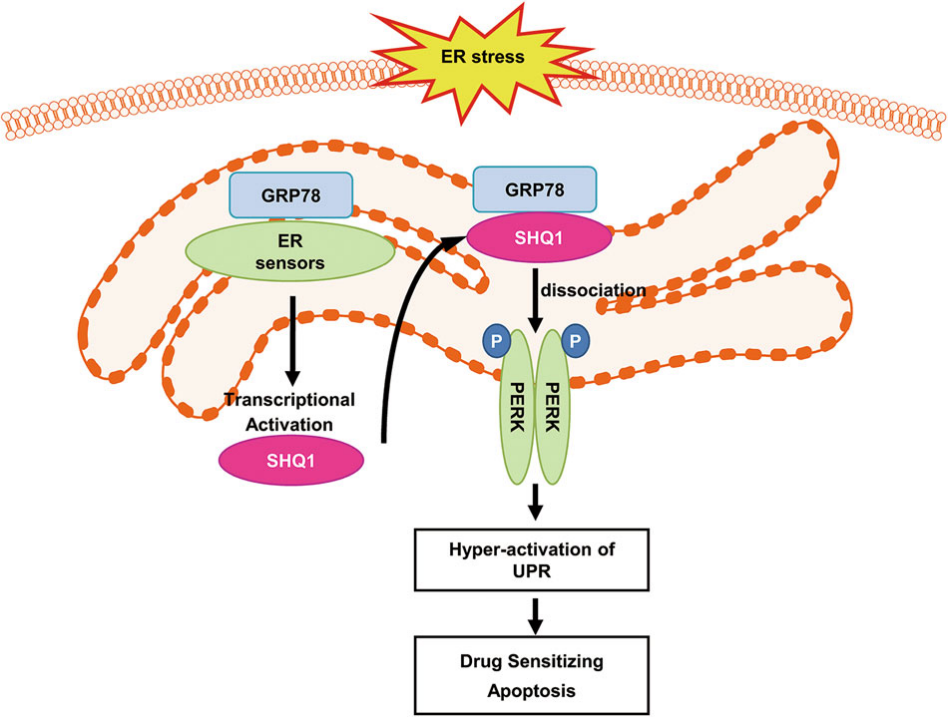
A schematic diagram illustrating the involvement of SHQ1 in ER-stress-mediated apoptosis. SHQ1 is an ER response gene that is transcriptionally activated by ER sensor signaling. SHQ1 interacts with the ER chaperone GRP78 to release ER sensors PERK/IRE1*α*/ATF6 from GRP78/ER-sensor complexes, leading to UPR hyper-activation, thereby triggering apoptosis and sensitizing chemotherapeutics-induced cell death.

In a summary, as an ER stress response gene, SHQ1 acted as a positive modulator of UPR and promoted UPR-mediated apoptosis (Fig. 6). In light of this, activation of SHQ1 might be a desirable approach for targeting some cancers.

## Supplementary information

a text summary of supplementary information

supplementary figure legends

supplementary figure 1

supplementary figure 2

supplementary figure 3

supplementary figure 4

## Data Availability

All data in this study are available upon request by contacting with the corresponding author.
