# Supplementary material for: SHQ1 is an ER stress response gene that facilitates chemotherapeutics-induced apoptosis via sensitizing ER-stress response
Source: Cell Death Dis. 2020 Jun 10;11(6):445. doi: 10.1038/s41419-020-2656-0 (PMC7286909; doi:10.1038/s41419-020-2656-0)
Supplement: Supplementary file 1 — a text summary of supplementary information [file 41419_2020_2656_MOESM1_ESM.docx]

The supplementary information consists of one supplemental figure legends, four supplementary figures and Author Contribution Form. The supplemental figure legends described detailed information about each supplementary figures. The signed Author Contribution Form was scanned and saved as Tiff format.
