# Supplementary material for: SHQ1 is an ER stress response gene that facilitates chemotherapeutics-induced apoptosis via sensitizing ER-stress response
Source: Cell Death Dis. 2020 Jun 10;11(6):445. doi: 10.1038/s41419-020-2656-0 (PMC7286909; doi:10.1038/s41419-020-2656-0)
Supplement: Supplementary file 2 — supplementary figure legends [file 41419_2020_2656_MOESM2_ESM.docx]

**Supplementary figure legends**

**Supplementary Fig. 1** SHQ1 is regulated by ATF6 and XBP1. **a-c.** ATF6-KD (shATF6-1, shATF6-2) HepG2 cells or XBP1-KD (shXBP1-1, shXBP1-2) HepG2 cells and control (pLKO.1) HepG2 cells were treated with 2.5 μg/ml TM as indicated for 24 h. The mRNA levels of ATF6, XBP1, spliced XBP1 (XBP1s) and SHQ1 were determined by qPCR. **d.** HEK293T cells were transfected with either individually or with a combination of Flag-ATF6 and HA-XBP1 plasmids and cultured for 24 h. Flag-EV, empty Flag vector. HA-EV, empty HA vector. The cells lysates were subjected to western blot analyses with indicated specific antibodies. Data shown are representative of three independent experiments. Quantified data represent the mean ± SD, data shown in (a)(b)(c) were analyzed by two-tailed unpaired Student’s *t* test.

**Supplementary Fig. 2** SHQ1 promotes apoptosis in MDA-MB-231 cancer cells. **a.** Establishment of SHQ1-knockout (SHQ1-KO) MDA-MB-231 cells and control MDA-MB-231 cells using CRISPR/Cas9 technology. Guide RNAs targeting the *SHQ1* gene were designed and transduced into MDA-MB-231 cells. Western blotting was used to confirm SHQ1 protein depletion in the genome-edited SHQ1 knockout MDA-MB-231 cells. **b.** SHQ1-KO MDA-MB-231 cells and control MDA-MB-231 cells were either treated or untreated with TM as indicated, the cell growth was measured using a MTT assay. The graph shows the cell growth across 7 days. The bar graph shows cell growth on the seventh day. **c.** The proportion of cells exhibiting apoptosis was measured using Annexin V/PI staining by Flow cytometry. **d.** The mRNA levels of CHOP were determined by qPCR at the indicated time points. **e.** The changes in apoptotic proteins were examined by western blotting at the indicated time points. C-PARP, cleaved PARP. C-Caspase 3, cleaved caspase 3. Data shown in this figure are representative of three independent experiments. Quantified data represent the mean ± SD, data shown in (**b**)(**c**) were analyzed by two-tailed unpaired Student’s *t* test, and the data shown in (**d**) were analyzed by two-way ANOVA analysis.

**Supplementary Fig. 3** SHQ1 deficiency leads to down-regulation of UPR related genes. **a.** The SHQ1-KO MDA-MB-231 cells and control MDA-MB-231 cells treated with or without 0.25 μg/ml TM for 20 h were cultured to 80% confluence, followed by RNA-seq analysis (n=3). Clustering heat maps of gene expressions in SHQ1-KO cells and control cells treated with or without TM. The cellular components of GO terms in which most changed downregulated genes enriched in SHQ1-KO cells treated with or without TM are shown. The negative log of padj value (base 10) is plotted on the X-axis. **b.** SHQ1-KO MDA-MB-231 cells and control MDA-MB-231 cells were treated with TM for the indicated time points, and the expression of ER sensors was monitored using western blotting. O and P indicate mobility changes for non-activated PERK and activated PERK by phosphorylation. p-PERK, phosphorylated PERK; p-IRE1α, phosphorylated IRE1α; p50ATF6, activated form of ATF6; p90ATF6, the full length precursor form of ATF6. **c.** SHQ1-KO MDA-MB-231 cells and control MDA-MB-231 cells were treated with TM as indicated, and qPCR was performed to evaluate the expression of UPR related genes (n = 3). Quantified data represent the mean ± SD, two-way ANOVA analysis. Data shown in this figure are representative of three independent experiments.

**Supplementary Fig. 4** SHQ1 increases the anti-tumor efficiency of CDDP against MDA-MB-231 cancer cells. **a, b.** SHQ1-KO MDA-MB-231 cells and control-MDA-MB-231 cells were treated with different doses of cisplatin (CDDP) as indicated. **a.** Cell growth was assessed via Real-Time Cell Analysis. The bar graph shows the normalized cell index at 70 h in each group. **b.** The proportion of cells exhibiting apoptosis was measured using Annexin V/PI staining by Flow cytometry after treating them with CDDP for 48 h. Data are representative of at least three independent experiments. Quantified data represent the mean ± SD, two-tailed unpaired Student’s *t* test.
