# Supplementary figures and images for: SHQ1 is an ER stress response gene that facilitates chemotherapeutics-induced apoptosis via sensitizing ER-stress response
Source: Cell Death Dis. 2020 Jun 10;11(6):445. doi: 10.1038/s41419-020-2656-0 (PMC7286909; doi:10.1038/s41419-020-2656-0)

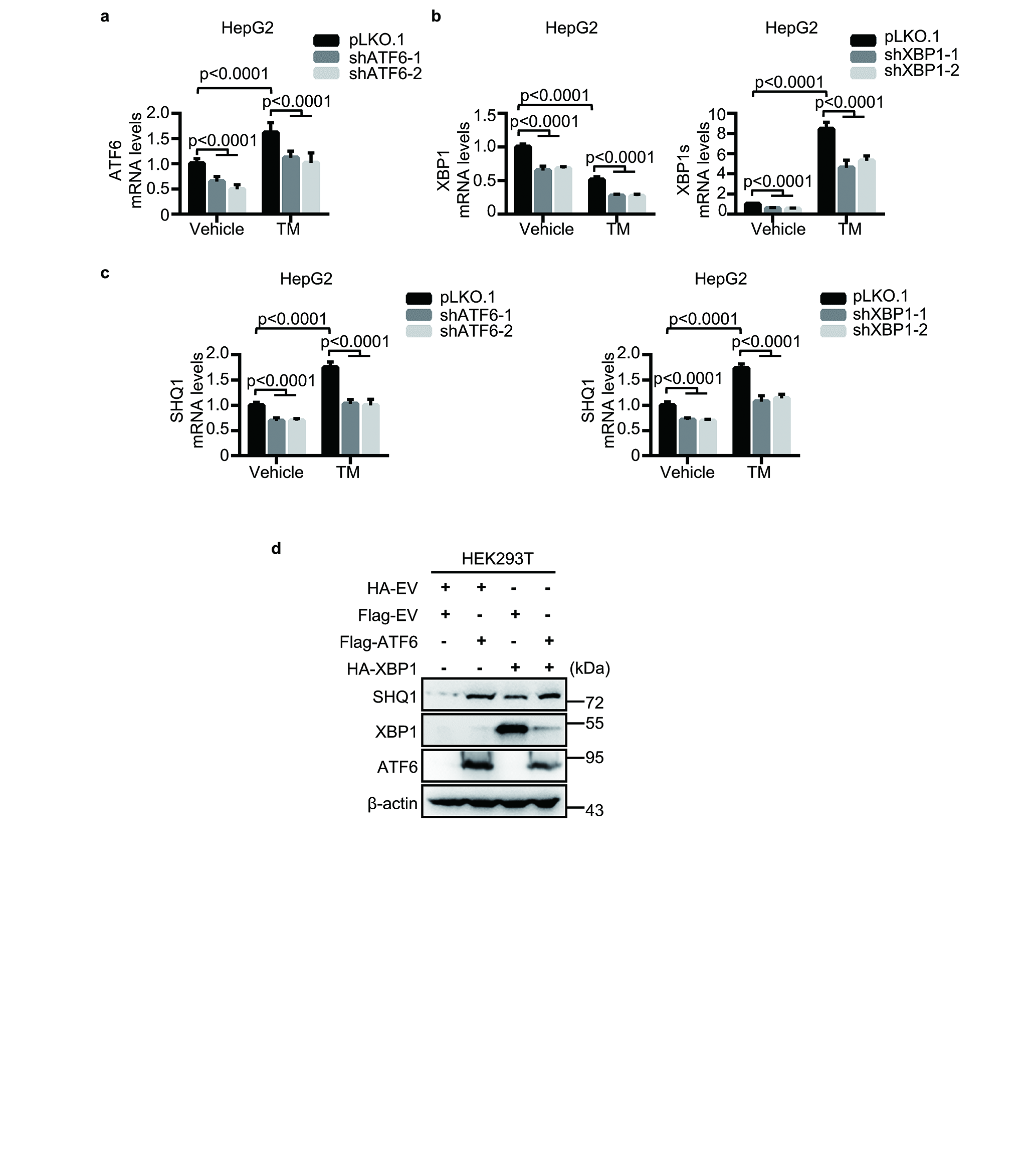

Supplement: Supplementary file 3 — supplementary figure 1 [file 41419_2020_2656_MOESM3_ESM.tif]

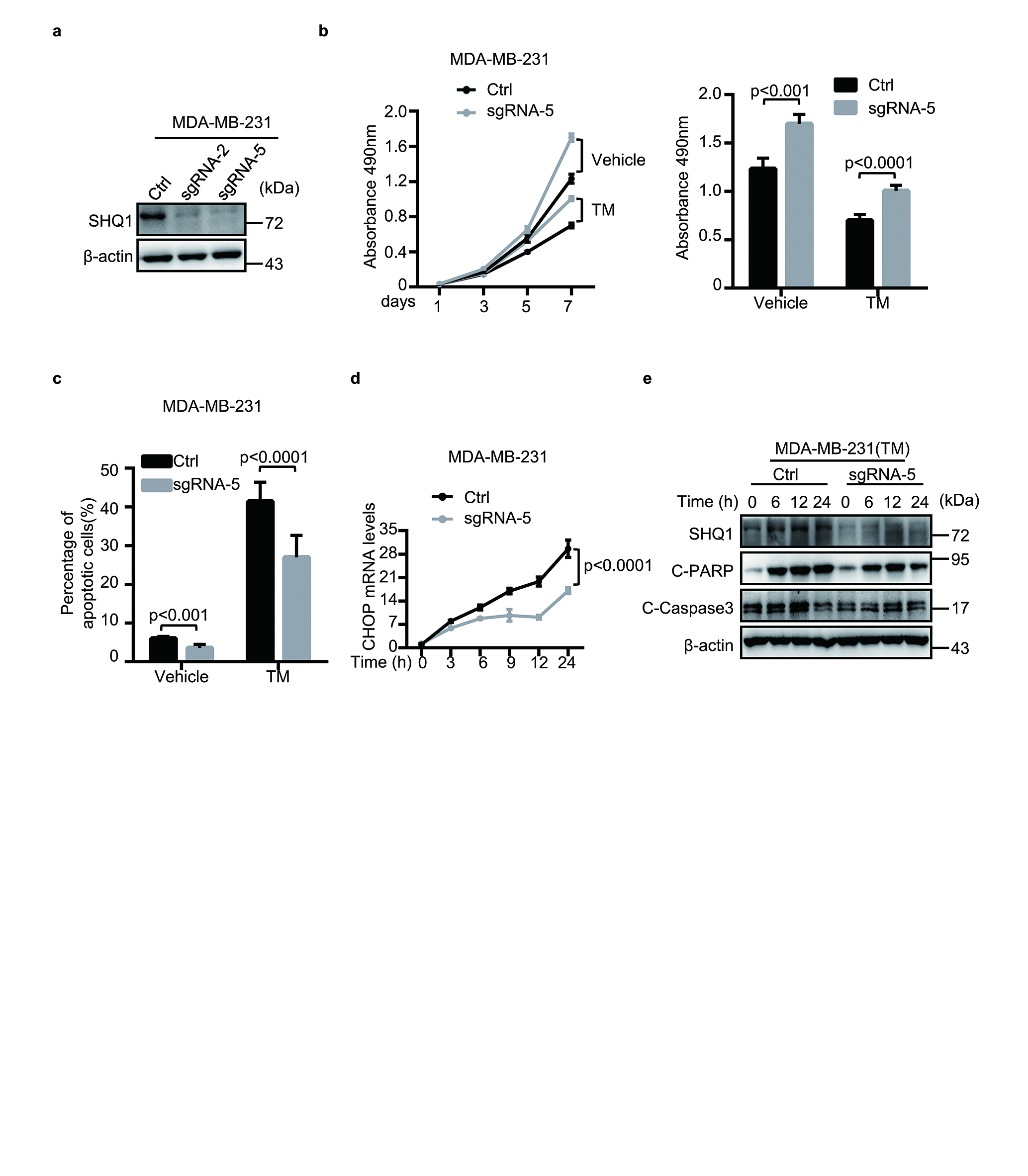

Supplement: Supplementary file 4 — supplementary figure 2 [file 41419_2020_2656_MOESM4_ESM.tif]

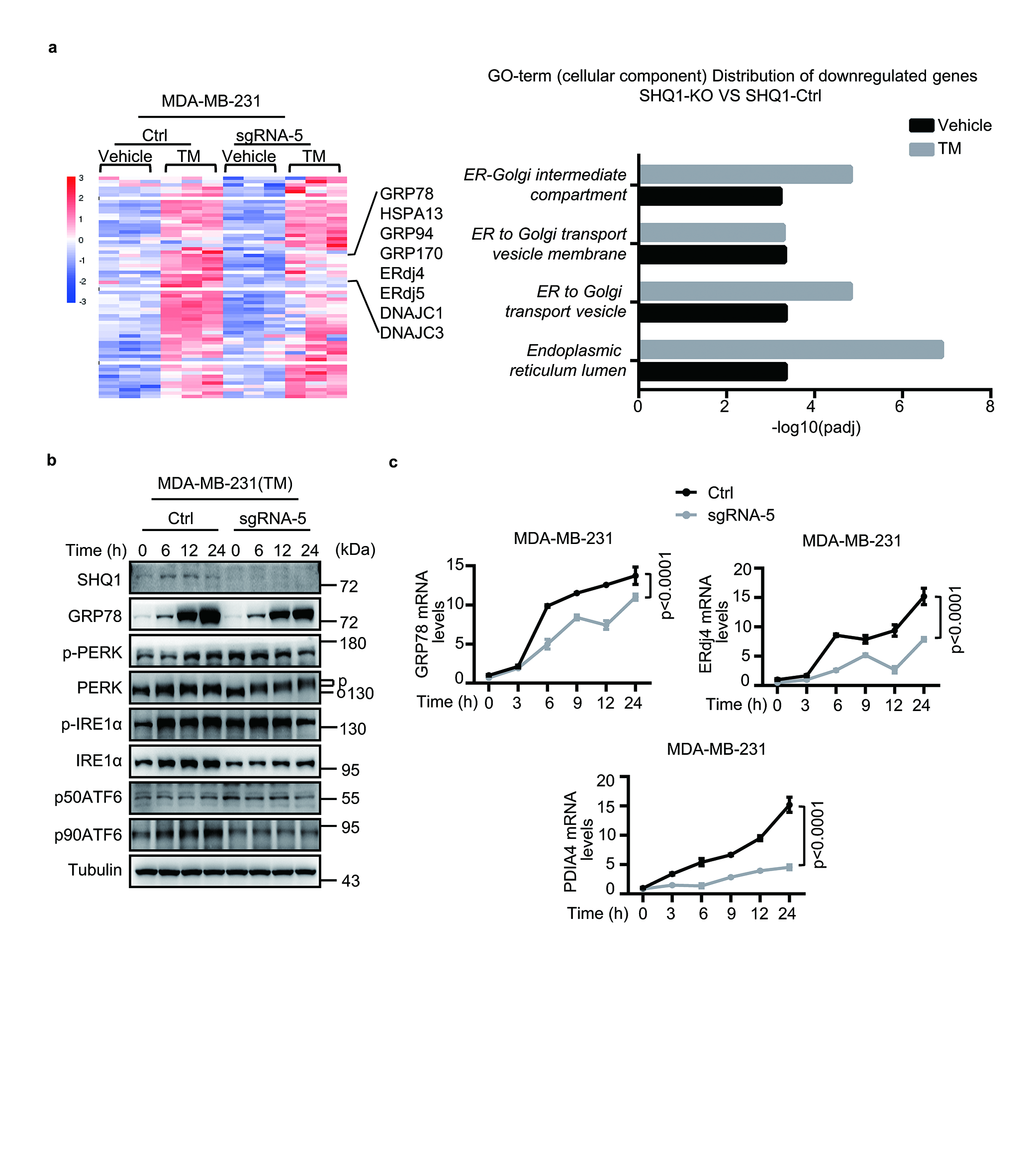

Supplement: Supplementary file 5 — supplementary figure 3 [file 41419_2020_2656_MOESM5_ESM.tif]

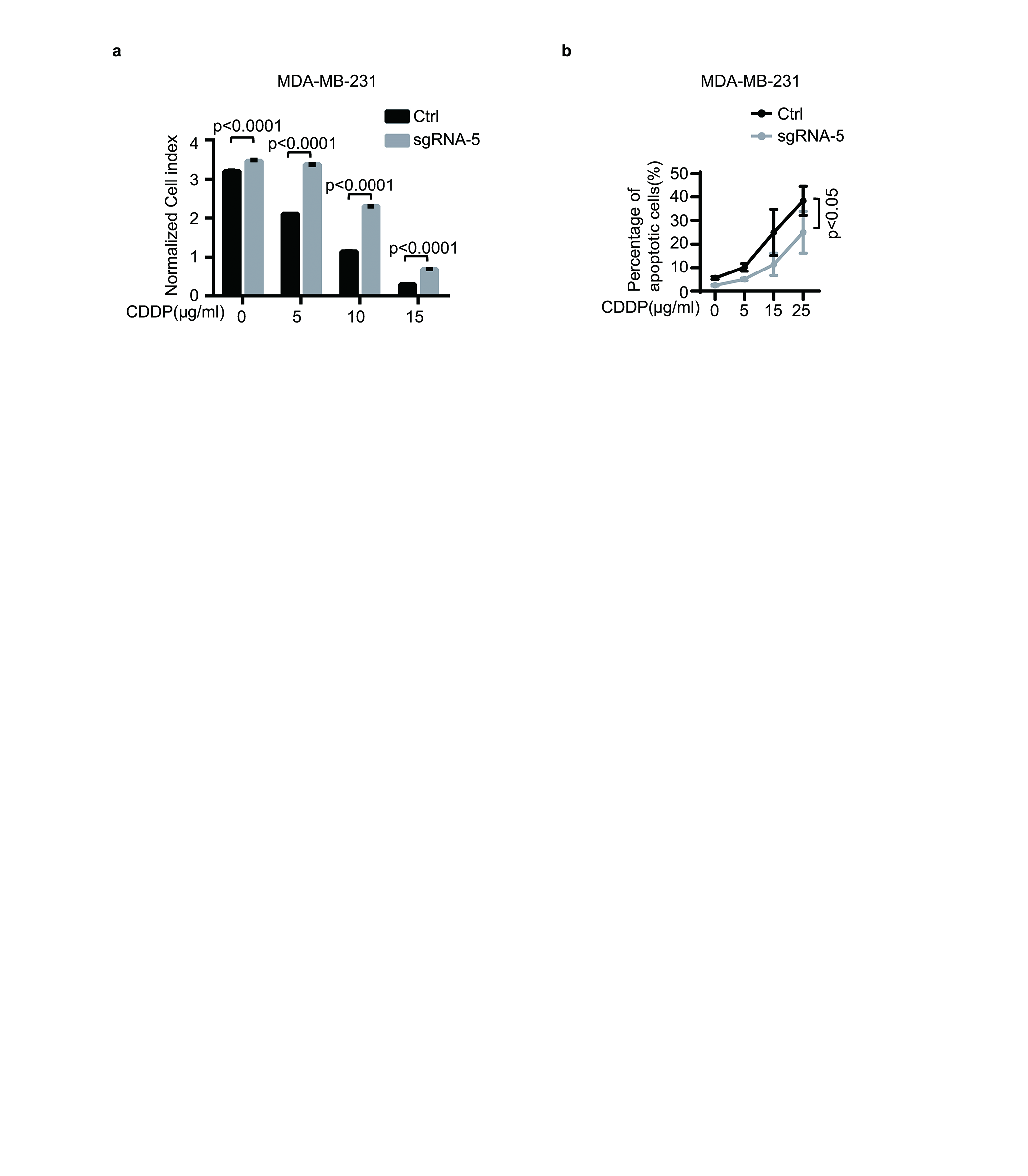

Supplement: Supplementary file 6 — supplementary figure 4 [file 41419_2020_2656_MOESM6_ESM.tif]
